# Supplementary material for: Site Density Functional Theory and Structural Bioinformatics Analysis of the SARS-CoV Spike Protein and hACE2 Complex
Source: Molecules. 2022 Jan 26;27(3):799. doi: 10.3390/molecules27030799 (PMC8839245; doi:10.3390/molecules27030799)
Supplement: Supplementary file 1 [file molecules-27-00799-s001.zip › molecules-1537188-supplementary.pdf]

Supplementary Materials: SITE DENSITY FUNCTIONAL THEORY COMBINED WITH BIOINFORMATICS METHODS FOR THE SARS-CoV-2 SPIKE AND hACE2 COMPLEX

Nitesh Kumawat, Andrejs Tucs, Soumen Bera, Gennady N. Chuev, Marat Valiev, Marina V. Fedotova, Sergey E. Kruchinin, Koji Tsuda, Adnan Sljoka and Amit Chakraborty

Table S1. Interface residual interactions between CoV-RBD and hACE2

	SARS-CoV-1-RBD-hACE2		SARS-CoV-2-RBD-hACE2	
	Receptor	RBD Domain	Receptor	RBD Domain
1	TYR83	ASN473	LYS353	GLY502
2	GLN24	ASN473	TYR41	THR500
3	ASP38	TYR436	TYR41	ASN501
4	GLU37	TYR491	ASP38	TYR449
5	LYS353	TYR481	GLU35	GLN493
6	LYS353	GLY482	THR27	TYR489
7	LYS353	GLY488		
8	TYR41	THR486		
9	GLU329	ARG426		

Table S2. Interface interactions between RBD and hACE2

	CoV-1-RBD-hACE2	CoV-2-RBD-hACE2
No. of intermolecular contacts	55	59
No. of charged-charged contacts		1
No. of charged-polar contacts	8	9
No. of charged-apolar contacts	19	18
No. of polar-polar contacts	2	4
No. of apolar-polar contacts	17	18
No. of apolar-apolar contacts	8	9
Percentage of apolar NIS residues	34.96	36.60
Percentage of charged NIS residues	27.36	35.43

Table S3. 3DRISM parameters of calculated complexes

complex	6m17	2ajf	6vyb
Charge of clipped part	1.0	-1.0	2.0
Total number of heavy atoms	25344	12777	22365
Number of heavy atoms of clipped part	1244	1272	492
Calculation box size (Å)	87×80×87	87×80×87	80×75×63

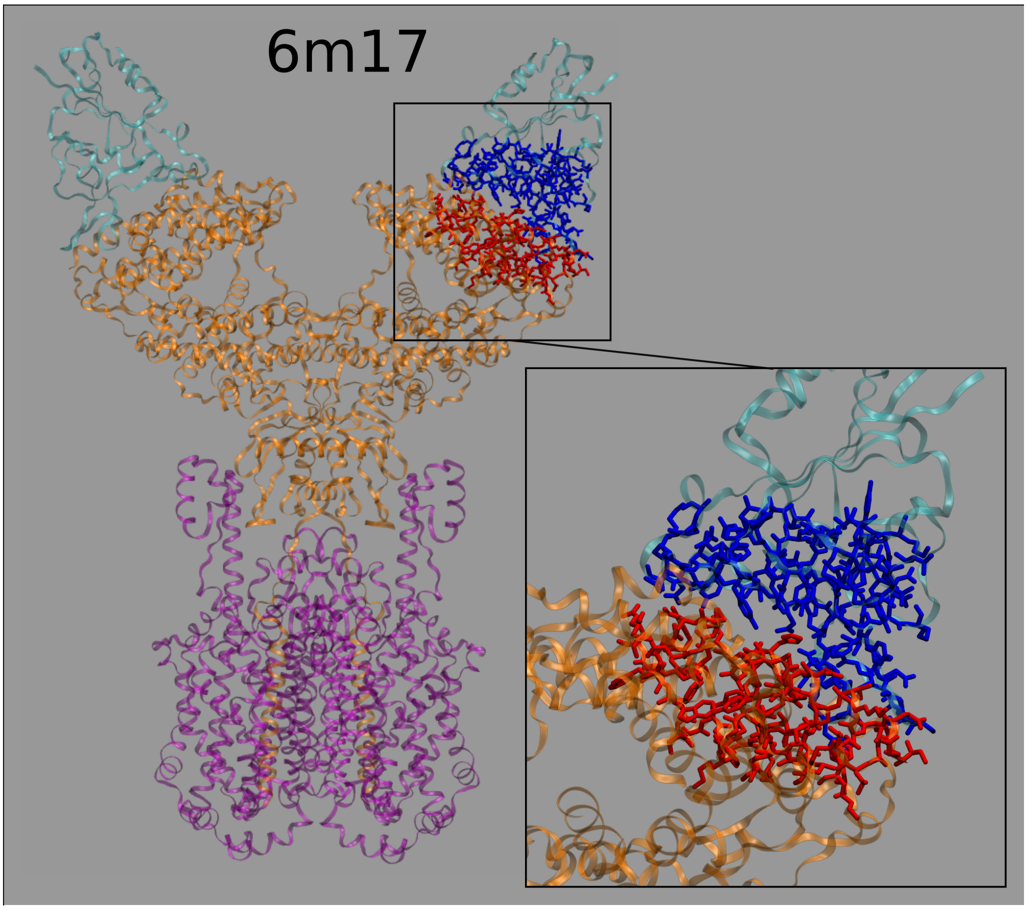

Figure S1. 6m17 complex. Purple ribbons - B(0)AT1 neutral amino acid transporter, orange ribbons - hACE2, cyan ribbons - surface spike of SARS-CoV-2, blue and red structures are parts of the spike and hACE2 used for 3DRISM calculations.

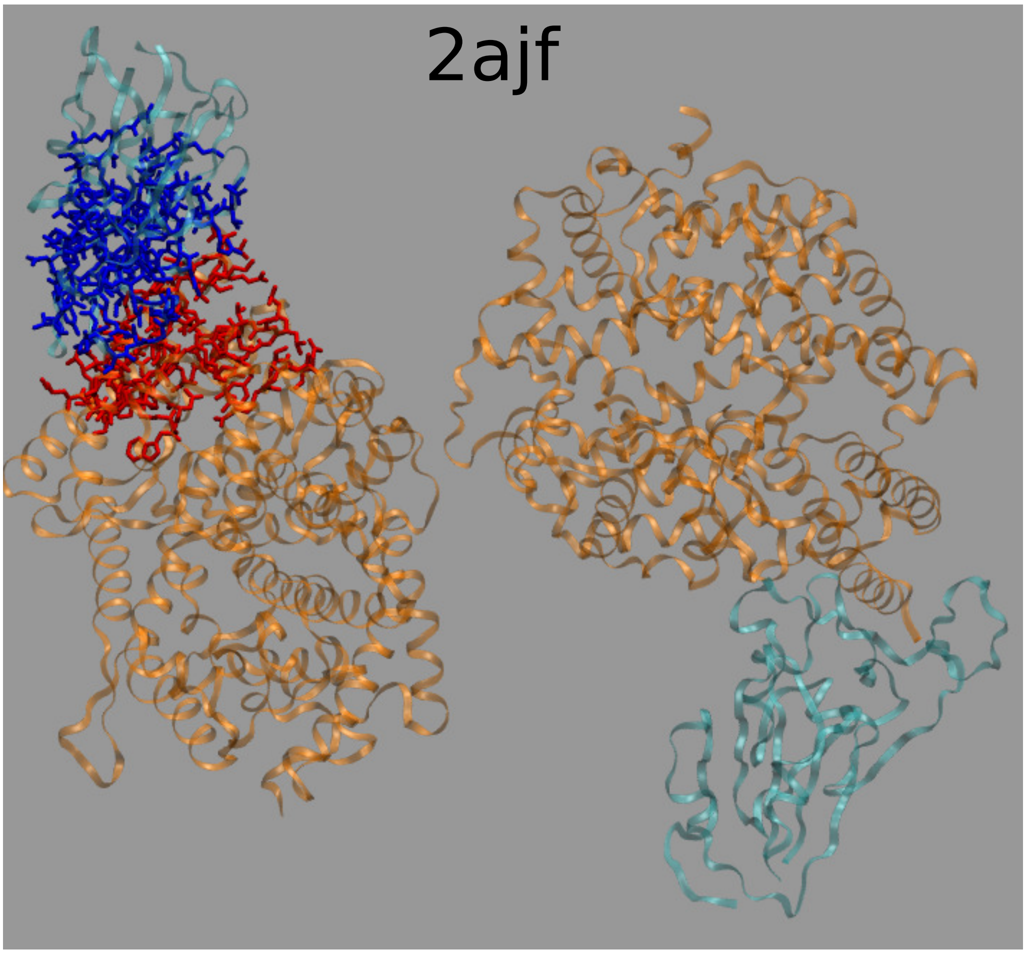

Figure S2. 2ajf complex. Orange ribbons - hACE2, cyan ribbons - spike of SARS-CoV, blue and red structures are parts of the spike and hACE2 used for 3DRISM calculations.

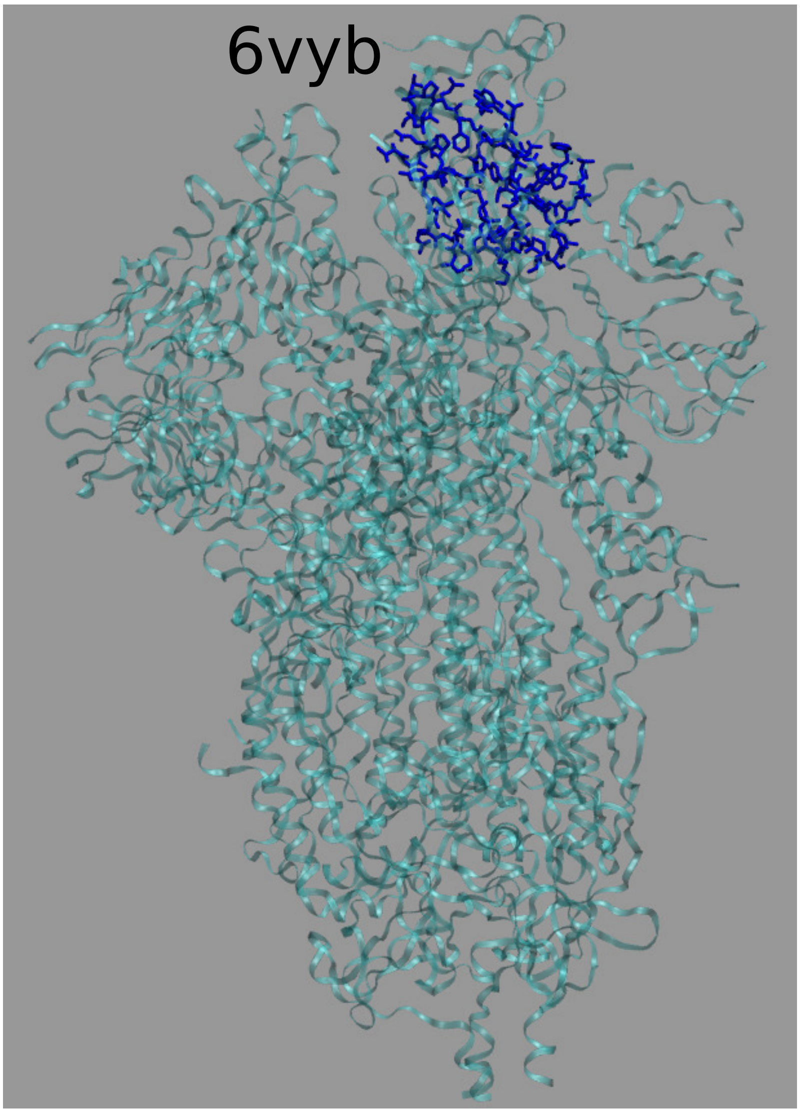

Figure S3. 6vyb complex. Cyan ribbons - spike of SARS-CoV-2, blue structure is a part of spike used for 3DRISM calculations

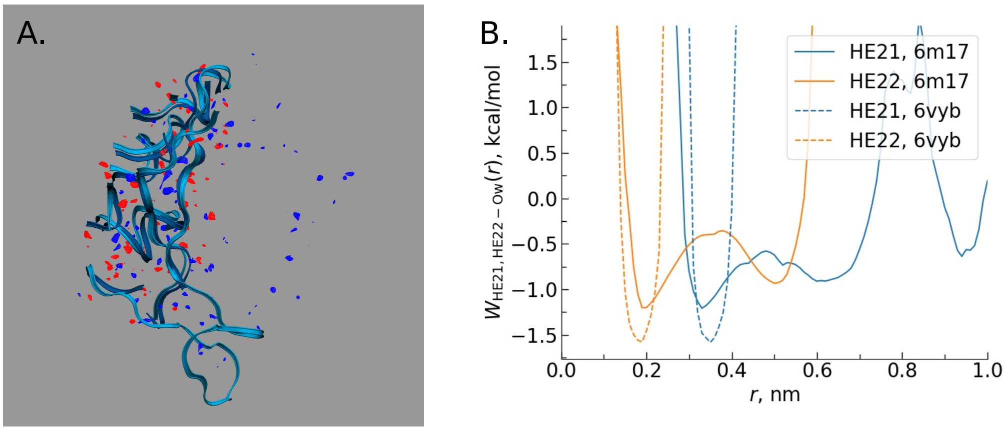

Figure S4. Water distribution around RBD of SARS-CoV-2. (**A**) Differences in distributions of water oxygen (blue) and water hydrogens (red) between the CoV-2/hACE2 and CoV-2. (**B**) The pmf of RBD-O and RBD-H for the CoV-2 and CoV-2/hACE2 complex. The RBD is indicated by blue ribbons, the RBD of CoV-2 is shown as background for the differences.

Table S4. Average RMSF values of the whole spike protein SARS-CoV-1 and SARSCoV-2 (top table). The corresponding values for the hACE2-RBD complex (bottom table). Highest value among SARS-CoV-1 and SARS-CoV-2 is indicated in bold.

	All domains		RBD up	
Cut-off (kcal/mol)	SARS-CoV-1	SARS-CoV-2	SARS-CoV-1	SARS-CoV-2
−1.0	**6.5±3.9Å**	5.9±4.7Å	17.7±5.6Å	**18.0±7.9Å**
−2.0	**8.7±5.6Å**	6.1±4.3Å	**20.1±6.2Å**	16.4±6.8Å
−3.0	**9.4±5.8Å**	6.5±4.4Å	**24.8±7.9Å**	16.6±6.8Å

	hACE2		RBD	
Cut-off (kcal/mol)	SARS-CoV-1	SARS-CoV-2	SARS-CoV-1	SARS-CoV-2
−1.0	3.5±1.1Å	**4.0±1.3Å**	**5.7±2.1Å**	4.8±1.9Å
−2.0	**4.4±1.4Å**	4.3±1.4Å	**7.0±2.9Å**	5.5±2.3Å
−3.0	**4.9±1.8Å**	4.7±1.4Å	**7.7±3.1Å**	6.3±2.5Å

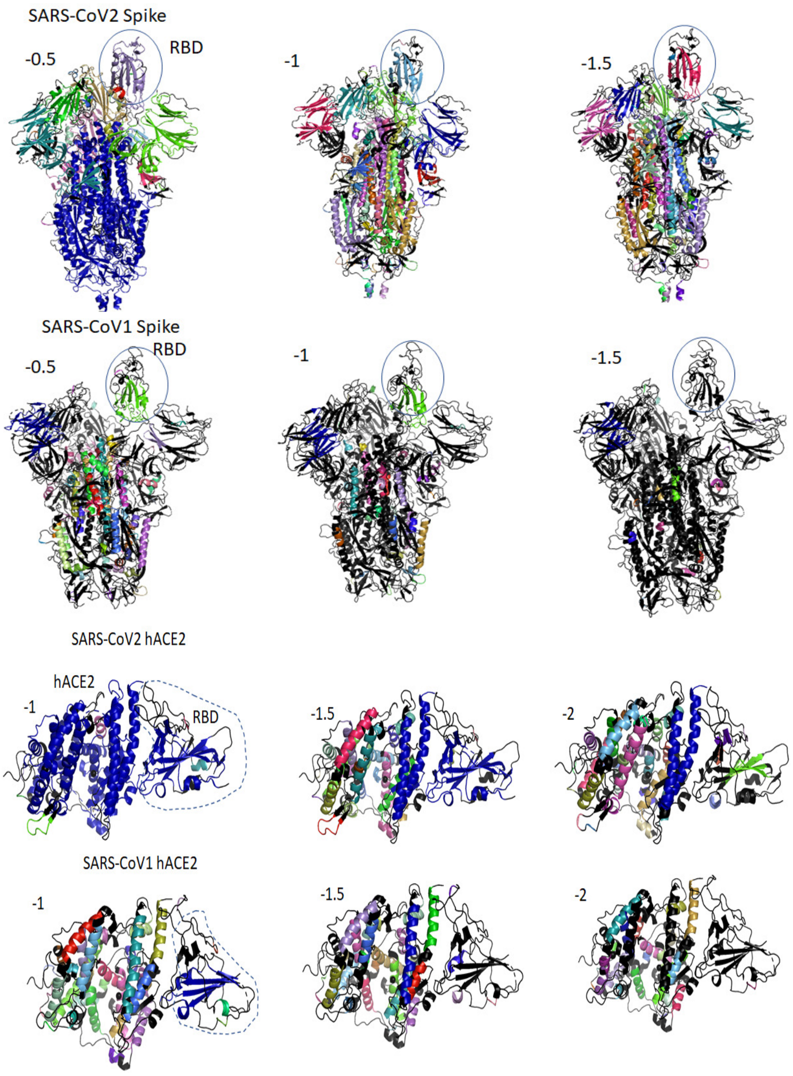

Figure S5. Rigidity analysis of protein structures. Individual colours indicate presence of large rigid clusters (blue being the largest rigid cluster), while black colour represents flexible regions.

Table S5. Summary of rigidity analysis

	Bound/unbound (PDB IDs)			
Energy cutoff (kcal/mol)	SARS-CoV-2 S (6vyb)	SARS-CoV-1 S (6crz)	CoV1-RBD ACE2 (2ajf)	CoV2-RBD ACE2 (6m0j)
degree of freedoms				
-0.1	3005	3744	823	716
-0.5	3468	5310	1002	818
-1.0	3881	6480	1204	923
-1.5	4279	7307	1371	1064
-2.0	4597	7897	1489	1246
-2.5	5075	8343	1642	1387
-3.0	5455	8684	1784	1535
rigid clusters				
-0.1	4502	7273	1223	1028
-0.5	6112	11797	2120	1337
-1.0	8224	13052	2560	1607
-1.5	9587	13681	2837	2275
-2.0	10259	13979	3015	2702
-2.5	10713	14042	3116	2894
-3.0	11261	14076	3199	3003
